# Threshold effect of technological innovation on carbon emission intensity based on multi-source heterogeneous data

**DOI:** 10.1038/s41598-023-46406-3

**Published:** 2023-11-04

**Authors:** Xiaochun Zhao, Huixin Xu, Shi Yin, Ying Zhou

**Affiliations:** 1 School of Management, Anhui University, Hefei, 230601 China; 2 College of Economics and Management, Hebei Agricultural University, Baoding, 071001 China

**Keywords:** Environmental sciences, Environmental social sciences, Mathematics and computing

## Abstract

It is of much importance to clarify the impact of technological innovation on carbon emission intensity for the low-carbon transformation of China's economy. This study, based on the panel data of 30 Chinese provinces and municipalities from 2010 to 2020, measures and analyzes the carbon emission intensity and the level of technological innovation, establishing a spatial econometric model to study the spatial spillover effect and a panel threshold model to analyze the nonlinear influence of technological innovation level on carbon emission intensity. The findings are as follows: First, the overall carbon emission intensity in China shows a decreasing trend from 2010 to 2020, with the average dropping from 3.09 in 2010 to 1.98 in 2020; Second, the spatial autocorrelation results reveal that the level of technological innovation and carbon emission intensity in China are obviously aggregated in the global spatial distribution pattern. Third, the regression results of the spatial econometric model show that the direct effect of technological innovation on carbon emission intensity is significantly negative at the level of 1%, that is, the improvement of the technological innovation in a certain area has a significant inhibitory effect on carbon emission intensity. Fourth, based on the level of economic development, there is a significant three-threshold effect of the level of technological innovation on carbon emission intensity in China, and the influence of the level of technological innovation on carbon emission intensity varies in the direction of existence and coefficient values within different threshold intervals. As economic development reaches the third interval, the technological innovation level has the most significant inhibition on carbon emission intensity. These findings enriches the research of the nonlinear relationship between technological innovation and carbon emission intensity, clarifies the spatial spillover effect and threshold effect between among them, and provides inspiration for better promote the low-carbon transformation of economy.

## Introduction

Since the first industrial revolution in human society, with the rapid development of productivity, the concentration of carbon dioxide in the atmosphere has been on the rise, which has threatened the global ecosystem, causing global warming to attract worldwide attention^1^. Global warming has led to glacier melting, sea level rise, and frequent occurrence of extreme weather, which has seriously threatened human survival. To this end, it has become a global consensus to reduce CO_2_ emissions and achieve green and low-carbon development of the world economy^2^. In order to cope with climate change, a series of binding conventions, such as the United Nations Framework Convention on Climate Change, the Kyoto Protocol, the Paris Agreement, and so on, have been reached, which clearly put forward the goals and objectives to be achieved in limiting CO_2_ emissions. However, these series of agreements have not been able to effectively curb the rising trend of global carbon emissions. Under the influence of COVID-19, global carbon dioxide emissions in 2020 will decrease by 5.9% compared with 2019. However, with the arrival of the post-epidemic era, the recovery of the global economy, and the sharp rebound of global energy demand, the global carbon emissions in 2021 will reach 33.884 billion tons, up by 5.6%. Therefore, countries have introduced relevant policies to achieve net-zero global CO_2_ emissions as soon as possible, intending to keep the global average temperature within 2 °C. As an active supporter and participant in global climate governance, China formally proposed the goal of achieving a carbon peak by 2030 and carbon neutrality by 2060 at the 75th United Nations General Assembly in September 2020^3^. In general, the carbon peaking and carbon neutrality goal, as the strategic starting point to achieve the Chinese path to modernization, is a must-answer question, not a multiple-choice question^4^. To achieve the goal of carbon peaking and carbon neutrality at an early date, the Chinese government has orderly promoted the work of carbon peaking and carbon neutralization. China will transition from assessing the total amount and intensity of energy consumption to assessing the total amount and intensity of carbon emissions, improve policy incentives for reducing pollution and carbon emissions and policy constraints on such emissions, and promote faster development of green ways of working and living.

The carbon peaking and carbon neutrality proposed by the Chinese government is an extensive and profound economic and social systematic change that will completely change the way of production and life of mankind. Scientific and technological innovation, as an important engine to support this transformation, plays an important role in it. According to the statistics of the International Energy Agency, about 80% of global carbon emissions come from the energy consumption necessary to support human modern life, mainly fossil energy^5^. At present, the prosperity of the world economy and the beauty of human life are at the cost of massive consumption of fossil energy and material resources. Research shows that scientific and technological innovation can improve the utilization efficiency of fossil energy and be available for exploration and use of new energy to a certain extent, thus significantly reducing carbon dioxide emissions^6^. The Chinese government must rely on scientific and technological breakthroughs and innovation if it wants to achieve low-carbon and zero-carbon goals, and to transform from fossil energy support to non-fossil energy. For this reason, the Chinese government pointed out that innovation is the first driving force to lead the development and the strategic support to build a modern economic system^7^. Therefore, studying the impact of technological innovation on carbon emission intensity is of great significance for China to achieve the carbon peaking and carbon neutrality goal and transform it to a low-carbon economy and society. In addition, the study of the impact of technological innovation on carbon emission intensity is conducive to providing inspiration for the Chinese government to formulate targeted scientific and technological innovation policies and carbon reduction policies, so as to give full play to the role of scientific and technological innovation in carbon reduction. The aim of this study is to explore the spatial correlation between technological innovation level and carbon emission intensity, as well as the nonlinear relationship.

The structure of this study is as follows: the first section is introduction, which introduces the necessity of exploring the impact of technological innovation on carbon emission intensity. Second section is literature review, exploring the research achievements of previous scholars from the perspective of the inhibitory or promoting effects of technological innovation on carbon emissions. Third section is model construction and variable description, this section constructs a spatial econometric model and a threshold regression model, and explains the data and sources selected in this article. Fourth section is analysis results, which explores the spatial spillover effects and threshold effects between technological innovation and carbon emission intensity through the analysis of empirical results. Fifth section is the conclusion and recommendations. Based on empirical findings, this section proposes some suggestions. Sixth section is research deficiencies and prospects, which explains the shortcomings in the research and corresponding solutions.

## Literature review

The concept of “low-carbon” development is prevalent, how to balance the relationship between economic development and carbon emissions has always been a hot topic of academic exploration^8,9^. Technological innovation has an increasing impact on carbon emissions^10,11^, which has also attracted many scholars to continuously explore the relationship between technological innovation and carbon emissions^12–14^.

There are two different views on the relationship between technological innovation and carbon emissions. Firstly, scholars believes that with the improvement of technological innovation level, there is a certain inhibitory effect on carbon emissions. This positive emission reduction effect stems from technological innovation that reduces carbon dioxide emissions by improving energy efficiency, saving costs, and enhancing various spillover effects^15–17^. Especially with human breakthroughs in new energy technologies, the utilization rate of clean energy fundamentally surpasses that of fossil fuels, thus achieving the goal of emission reduction. Mcqueen, et al. pointed out that developing technologies such as low-temperature heat sources can reduce the cost of direct air capture (DAC), thereby incentivizing businesses to compress and directly use the emitted carbon dioxide, thereby achieving the goal of reducing carbon emissions^18^. He et al. explored the impact of low-carbon technology innovation on carbon emissions based on data from 25 provinces in China from 2002 to 2015, and found that low-carbon technology is beneficial for improving carbon emission efficiency, reducing carbon emissions, and achieving sustainable development^19^. Suki et al. used a bootstrap autoregressive distribution lag (BARDL) model to examine the relationship between environmental degradation indicators, and the final research results showed that technological innovation can promote the consumption of renewable energy and reduce carbon emissions^20^.

Secondly, another scholars proposed that with the development of technological innovation, regional carbon emissions may significantly increase^21^. For this situation, Berhout et al. believe that it is due to the existence of the “rebound effect” of energy, that is, although the improvement of technological innovation can improve energy efficiency and save resources, it may reduce the unit production cost and price of products while improving energy efficiency, leading to an increase in product demand and consumption. Ultimately, the energy saved due to the improvement of technological innovation level is offset by the energy consumed by additional consumption^22^. Jiao et al.^23^ also demonstrated through empirical analysis that the increase in exports and technological innovation does indeed lead to an increase in carbon emissions. In addition, Adebayo et al. found through research on the sustainability impact of technological innovation on East Asia and the Pacific that technological innovation can promote economic development to a certain extent, but it can also cause significant energy consumption in the short term, thereby increasing carbon emissions to a certain extent^24^. In fact, many countries in the world currently rely on the consumption of fossil fuels to develop their economies^25^. For these countries, technological innovation can only enable them to better develop and use energy, leading to a surge in carbon emissions^26^. Therefore, such views believe that advanced technology will indeed reduce energy consumption intensity to a certain extent, but ultimately due to the existence of the “rebound effect”, the improvement of technological innovation leads to a continuous increase in output levels, leading to an increase in energy consumption and total carbon emissions^27^.

In summary, scholars have made considerable achievements in the research on the relationship between technological innovation and carbon emissions, but there is still room for improvement in the existing literature. Firstly, some studies on the impact of technological innovation on carbon emissions have not yet reached a unified conclusion. Secondly, the existing literature is more concerned with the impact of technological innovation on total carbon emissions rather than the impact on the carbon intensity, which is a better reflection of the development of a low-carbon economy as the CO_2_ emissions per unit of GDP growth^28^. Thirdly, there is little literature analyzing the relationship between technological innovation and carbon emissions from a spatial perspective, while most analyzing the linear relationship between technological innovation and carbon emissions, ignoring the nonlinear relationship between the two due to the existence of spillover effects and threshold effects^29^. This paper first selects appropriate indicators to measure and analyze the carbon emission intensity of all provinces, regions, and cities in China, and then constructs a spatial measurement model to explore the spatial correlation of technological innovation to carbon emission intensity. The control variables such as R&D investment intensity and higher education penetration rate and threshold variable as economic development level have been added to explore the nonlinear relationship between technological innovation and carbon emission intensity, which will provide more basis for decision-making departments to formulate energy conservation and emission reduction policy.

In summary, this paper addresses two research gaps in existing studies. Firstly, previous studies mainly analyzed the impact of technological innovation on total carbon emission, without focusing on the impact of technological innovation on carbon emission intensity. This paper analyzes the impact of technological innovation on carbon emission intensity. Secondly, the existing studies did not analyze the impact of technological innovation on carbon emission from a spatial perspective. This paper adopted exploratory spatial data analysis to analyze the impact of technological innovation on carbon emission intensity.

## Model construction and variable description

### Calculation of carbon emission intensity

The calculation of carbon emissions is based on the carbon emission coefficient given by the United Nations Intergovernmental Panel on Climate Change (IPCC), as well as the consumption of coal, coke, crude oil, natural gas, and other eight kinds of energy collected in various regions. In addition, since the carbon emission coefficient of water power, wind power, light energy, and other resources is 0, there are basically no problems related to carbon emissions, for which they are excluded from the calculation^30^. At present, there are two methods for measuring carbon dioxide emissions in academia: one is the material balance method, and the other is the coefficient method. This paper uses the material balance algorithm to calculate the carbon emissions of each region. Its formula refers to the research of Yu et al.^31^ and other scholars and the carbon emission intensity is the ratio of the total regional carbon emissions to the gross regional product (GDP).

1 $$C = \sum\limits_{t = 1}^{8} {C_{t} } = \sum\limits_{t = 1}^{8} {E_{t} } \times SCC_{t} \times CEC_{t} \times \frac{44}{{12}}$$

2 $$c = \frac{C}{GDP}$$

Where, *C* is the total carbon emission;* E* is the energy consumption; *SCC* is the conversion coefficient of standard coal; *CEC* is the carbon emission coefficient specified by IPCC; *c* is the carbon emission intensity, the unit of carbon emission intensity is ton/ten thousand yuan.

### Exploratory spatial data analysis

#### Global spatial autocorrelation

The global Moran'I value is used to analyze the overall spatial association degree, and the formula is as follows:

3 $$I = \frac{{n\sum\nolimits_{i = 1}^{n} {\sum\nolimits_{j = 1}^{n} {W_{ij} } \left( {x_{i} - \overline{x} } \right)\left( {x_{j} - \overline{x} } \right)} }}{{\sum\nolimits_{i = 1}^{n} {\sum\nolimits_{j = 1}^{n} {W_{ij} \sum\nolimits_{i = 1}^{n} {\left( {x_{i} - \overline{x} } \right)^{2} } } } }}$$

In formula 3, *n* represents the province and city under study, *X*_*i*_ represents the carbon emission intensity or the level of technological progress of provinces and cities; *X*_*j*_ represents the carbon emission intensity or level of technological innovation of Province* j*, which is adjacent to Province *i*; *W*_*ij*_ is the spatial weight matrix. The value range of Moran'I index is between (  − 1, 1). If it is less than 0, it indicates a negative correlation. If it is greater than 0, it indicates a positive correlation. If it equals 0, it indicates that each spatial object unit in the study area is independent of the other. The closer *I* value is to 1, the more significant the agglomeration effect of a certain attribute of the research object in spatial distribution is. The closer the *I* value is to − 1, the more significant the dispersion effect of a certain attribute of the research object on the spatial distribution is.

#### Local spatial autocorrelation

In the actual spatial data distribution, when the amount of data is too large, the variable data in the local region often appears to be locally unstable due to the randomness of the data. Therefore, it is necessary to introduce the local spatial autocorrelation index to achieve the autocorrelation evaluation of the local region and reveal the spatial heterogeneity of the data. On the spatial position *i*, the local Moran'I index *I*_*i*_ is defined as:

4 $$I_{i} = \frac{{n\left( {x_{i} - \overline{x} } \right)\sum\nolimits_{j} {W_{ij} \left( {x_{j} - \overline{x} } \right)} }}{{\sum\nolimits_{i} {\left( {x_{i} - \overline{x} } \right)^{2} } }}$$

In formula (4), *I*_*i*_ represents the Moran'I index of specific province* i*; *X*_*i*_ represents the carbon emission intensity or the scientific and technological levels of province *i*, and *X*_*j*_ represents the carbon emission intensity or technological innovation level of* j* province adjacent to province *i*. *W*_*ij*_ is the spatial weight matrix. When* I*_*i*_ > 0, it means that the attribute value of the space unit is similar to that of the adjacent unit (high high or low low); When *I*_*i*_ < 0, it means that the attribute value of the space element is not similar to that of the adjacent element (high low or low high).

#### Measurement model construction

Common spatial econometric models include the spatial autoregressive model, spatial autocorrelation model, and spatial Durbin model. Equations (5), (6), and (7) are the spatial autoregressive model, spatial autocorrelation model, and spatial Durbin model, respectively. At the same time, to reduce heteroscedasticity, some variables were logarithmized. The construction of each spatial measurement model is as follows:

5 $$c_{it} = \alpha_{1} Wc_{it} + \beta_{1} Inpatent_{it} + \gamma_{x} InX_{it} + \mu_{i} + \varepsilon_{it}$$

6 $$c_{it} = \alpha_{1} Wc_{it} + \beta_{l} Inpatent_{it} + \gamma_{x} InX_{it} + \mu_{i} + v_{it} ,v_{it} = \theta Wv_{it} + \varepsilon_{it}$$

7 $$c_{it} = \alpha_{1} Wc_{it} + \beta_{l} Inpatent_{it} + \gamma_{x} InX_{it} + \vartheta_{1} WInts_{it} + \vartheta_{x} WInX_{it} + \mu_{i} + \varepsilon_{it}$$

This paper chooses the SDM model because it is a combination of spatial lag model and spatial error model, which can be established by adding corresponding constraints to the spatial lag model and spatial error model. Its advantage lies in considering both the spatial correlation of the dependent variable and the spatial correlation of the independent variable. The SDM model is indeed more suitable for this study. In addition, the SDM model can also analyze in detail the full effect of explanatory variables, which can be divided into direct effects and indirect effects according to their sources. These two can not only be used to reflect the influence of the explanatory variable on the dependent variable, but also to reflect the feedback effect caused by the influence of the independent variable on the dependent variable in adjacent regions. In formula (5, 6 and 7): *c*_*it*_ is the logarithm of the carbon emission intensity of province, region and city in year *t*; *Inpatent*_*it*_ is the logarithm of the scientific and technological levels of province, district and city in year *t*; *InX*_*it*_ is the logarithm of a series of control variables; *W* represents the spatial weight matrix; *α*, *β*, *θ* and* γ* are constant terms;${\mu }_{i}$ represents individual effect; ${\varepsilon }_{it}$ represents a random disturbance term.

#### Construction of threshold model

Hansen's method is used for reference to build a panel threshold model^32^. The advantage of the threshold model is that it can search for its threshold value from the sample data, divide it into reasonable intervals, and then judge the nonlinear relationship between the independent and dependent variables through the regression results of the threshold model. Therefore, this study selects this models to explore the impact of technological innovation on carbon emission intensity more comprehensively. At the same time, to reduce the heteroscedasticity of the data, all variables are logarithmic.

Single effect threshold model:

8 $$\begin{aligned} c_{it} = & \mu_{i} + \beta_{1} Inpatent_{it} \times I(Inpgdp \le \gamma_{1} ) + \beta_{2} Inpatent_{it} I(Inpgdp > \gamma_{1} ) \\ & \quad + \gamma_{x} InX_{it} + \varepsilon_{it} \\ \end{aligned}$$

Double threshold effect model:

9 $$\begin{aligned} c_{it} = & \mu_{i} + \beta_{1} Inpatent_{it} \times I(Inpgdp \le \gamma_{1} ) + \beta_{2} Inpatent_{it} I(\gamma_{1} < Inpgdp \le \gamma_{2} ) \\ & \quad + \beta_{3} Inpatent_{it} I(Inpgdp_{it} > \gamma_{2} ) + \gamma_{x} InX_{it} + \varepsilon_{it} \\ \end{aligned}$$

In formulas 8 and 9, *I *(*·*) is a characteristic function. When the conditions in brackets are met, the value is 1, otherwise, the value is 0; *γ*_1_, *γ*_2_ is the threshold value to be estimated. *Inpatent* is the logarithm of the explanatory variable, and *Inpgdp* is the logarithm of the threshold variable; The remaining variables, the same as those in the above spatial econometric model, will not be repeated.

### Variable description

The explained variable set in this paper is carbon emission intensity, with the level of technological innovation set as the core explanatory variable and the level of economic development set as threshold variable. The control variables include the intensity of R&D investment^33^, the level of national education^34^, the level of opening up^33^, the level of urbanization^35^, and the total energy consumption^36^. All variables are in Table1, the specific description of each variable is as follows:

- Carbon intensity(c): Carbon intensity refers to the carbon dioxide emissions per unit of GDP growth. This variable is mainly used to measure the relationship between a country's economic growth and carbon emissions, and carbon emissions, as a product of economic development, can intuitively express the relationship between them with carbon emission intensity. At the same time, this paper refers to the research of Zhang et al.^37^ to measure the impact of carbon emissions on the economy and society with carbon emission intensity, and takes this as the research object
- Level of technological innovation(Inpatent): technological innovation refers to innovation for the purpose of creating new technologies or innovation based on scientific and technological knowledge and the resources it creates, including innovation in materials, products, processes, means, etc. The level of technological innovation in this paper draws on the research of Liu et al.^38^ which refers to the narrow concept of measuring by the number of invention patents, that is, the index measured by the number of valid invention patent applications at the end of the year
- R&D investment intensity(rd): Usually refers to the proportion of enterprise R&D expenditure to the total operating income of the enterprise or the proportion of regional R&D investment to the regional GDP, and scientific and technological development is driven by a huge economy. Therefore, based on the research of Yu et al.^33^ this paper selects R&D investment intensity as one of the control variables
- National education level(Inhedu): To a certain extent, the level of education reflects the degree of civilization of society and the degree of attention to the development of science and technology, so this paper selects the national education level as the control variable. Drawing on Molthan-Hill et al.^34^ the number of graduates of this college is measured
- Economic development level(pgdp): A certain level of economic development can have a negative effect on society, not only affecting carbon emissions, but also affecting the level of technological innovation. At the same time, this paper refers to the research of Wu et al.^39^ and selects the level of economic development as the threshold variable. And measured in terms of GDP per capita
- Level of opening up(trade): The level of opening up can reflect the economic development of the region to a certain extent, and indirectly affect the improvement of technological innovation. Therefore, this paper draws on the research of Yu et al.^33^ as a control variable and measures the proportion of regional merchandise imports and exports as a share of GDP
- Urbanization level(urban): The urbanization level refers to the proportion of the urban population to the total population. Sun et al.'s research shows that an increase in urban population will have an impact on carbon emissions to some extent, and will also increase the level of technological innovation to some extent. Therefore, this paper uses it as a control variable and measures the level of urbanization in terms of the proportion of urban population to the total population ^35^
- Total energy consumption(Intce): As one of the control variables, it reflects the carbon emissions at the intuitive level. This paper refers to the research of Xu et al.^36^ to calculate the total energy consumption by converting the sum of various energy sources consumed in the region into standard coal.

**Table 1:** Variable description.

Classification	Variable	Symbol	Variable description
Interpreted variable	Carbon emission intensity	c	Calculated from formula (2)
Core explanatory variables	Technical innovation level	Inpatent	The number of valid local patent applications
Threshold variable	Economic development level	pgdp	Measured by local GDP per capita
Control variable	National education level	Inhedu	Measured by the number of college graduates
	National education level	rd	Measured by the proportion of scientific research funds in GDP
	Urbanization level	Urban	Measured by the proportion of urban population in the total regional population
	Total energy consumption	Intce	Measured by the sum of all kinds of energy consumed by the region
	Opening level	Trade	Measured by the proportion of the total import and export of regional commodities to GDP

### Data source

Due to the severe lack of data in Tibet and Hong Kong, Macao, and Taiwan regions, this study focuses on 30 provinces and cities in China, with the time range of 2010–2020 as the original data. The data in this study are all sourced from the China Statistical Yearbook, China Energy Statistical Yearbook, and China Science and Technology Statistical Yearbook from 2010 to 2021. All statistical yearbooks are published by the Chinese government. In this study, all the collected data and calculation results are uniformly reserved for two decimal places. At the same time, in order to prevent the phenomenon of heteroscedasticity in the calculation process, the logarithmic processing of some index data with maximum or minimum value in the collected data is carried out. In this paper, smoothing index method is used to complete some missing data in the index.

## Empirical analysis

### Calculation of carbon emission intensity

In order to understand the change in carbon emission intensity in various provinces and cities in China, the analysis is carried out from 2010 to 2020 as an example. Read Table 2 for details.

**Table 2:** The carbon emission intensity of provinces and cities in China from 2010 to 2020.

Regions	Years provinces										
	2010	2011	2012	2013	2014	2015	2016	2017	2018	2019	2020
Eastern regions											
Beijing	0.88	0.71	0.63	0.50	0.47	0.40	0.32	0.28	0.26	0.22	0.19
Tianjin	1.95	1.74	1.53	1.40	1.23	1.13	0.97	0.92	0.92	1.23	0.97
Hebei	3.94	3.71	3.46	3.23	2.97	3.05	2.83	2.65	2.55	2.60	1.62
Liaoning	2.71	2.43	2.22	1.95	1.87	1.79	2.28	2.23	2.09	2.21	2.60
Shanghai	1.67	1.52	1.40	1.35	1.21	1.14	1.05	0.95	0.91	0.78	0.41
Jiangsu	1.58	1.54	1.41	1.29	1.16	1.1	1.02	0.92	0.82	0.8	0.62
Zhejiang	1.27	1.15	1.05	0.96	0.91	0.85	0.76	0.72	0.65	0.57	0.66
Fujian	1.36	1.34	1.18	1.03	0.95	0.84	0.72	0.68	0.70	0.61	0.65
Shandong	2.38	2.17	2.06	1.77	1.72	1.77	1.67	1.55	1.40	1.52	1.78
Guangdong	0.96	0.95	0.88	0.78	0.72	0.66	0.61	0.58	0.54	0.48	0.53
Hainan	1.09	1.06	1.02	0.95	0.86	0.82	0.72	0.70	0.68	0.61	0.58
Central regions											
Shanxi	7.27	6.55	6.33	6.27	6.36	7.27	7.04	6.23	6.15	6.38	6.22
Jilin	3.25	3.01	2.64	2.31	2.17	1.75	1.67	1.60	1.63	2.13	1.55
Heilongjiang	3.26	2.86	2.77	2.55	2.41	2.35	2.27	2.23	2.20	2.84	2.48
Anhui	2.68	2.27	2.14	2.02	2.04	1.96	1.77	1.62	1.51	1.27	1.03
Jiangxi	3.46	3.01	2.81	2.71	2.72	2.73	2.72	2.73	2.73	2.51	2.50
Henan	2.62	2.47	2.06	1.89	1.78	1.60	1.43	1.28	1.18	0.97	0.81
Hubei	2.15	2.03	1.82	1.38	1.23	1.08	0.97	0.89	0.84	0.76	0.67
Hunan	2.54	2.21	1.93	1.71	1.54	1.44	1.36	1.29	1.26	1.13	1.11
West regions											
Nei Monggol	6.77	6.59	6.21	5.56	5.38	5.35	5.40	6.33	6.79	7.71	8.25
Guangxi	1.94	1.76	1.65	1.47	1.36	1.21	1.17	1.16	1.12	1.14	1.15
Chongqing	1.98	1.78	1.52	1.16	1.22	0.99	0.89	0.84	0.76	0.70	0.70
Sichuan	1.75	1.46	1.35	1.26	1.14	0.87	0.78	0.66	0.59	0.54	0.54
Guizhou	5.03	4.49	4.08	3.58	3.01	2.66	2.48	2.25	2.01	1.82	1.76
Yunnan	4.19	3.48	3.15	2.70	2.30	1.97	1.74	1.58	1.47	1.16	1.12
Shaanxi	3.06	2.82	2.77	2.64	2.53	2.46	2.36	2.16	1.89	1.95	2.00
Gansu	3.18	3.01	2.81	2.59	2.46	2.38	2.17	2.07	1.98	1.89	1.87
Qinghai	2.82	2.43	3.10	3.08	2.61	2.32	2.64	2.47	2.07	1.96	1.91
Ningxia	11.33	11.67	10.58	9.98	9.68	9.12	8.18	8.97	9.12	9.60	9.45
Xinjiang	3.51	3.48	3.72	3.89	3.93	4.22	4.32	4.13	3.89	3.76	3.78

It can be seen from Table 2 that the carbon emission intensity in most regions of China has declined by 2020. The carbon emission intensity of 11 provinces and cities, including Beijing, Shanghai, Zhejiang, and Jiangsu, has dropped to below 1. The carbon emission intensity of 10 provinces and cities, including Tianjin, Anhui, and Shandong, is between 1 and 2. The carbon emission intensity of Hebei, Liaoning, Jilin, Heilongjiang, and Jiangxi is between 2 and 3. The carbon emission intensity of Shanxi, Inner Mongolia, Ningxia, and Xinjiang is higher than 3. The above significant changes show that the carbon emission intensity of provinces and cities in China's region generally presents a spatial pattern of low in the south and high in the north, which is basically consistent with the current level of economic development among regions in China. The carbon emission intensity of Jiangsu, Zhejiang, Shanghai, and Beijing is lower than that of most central and western regions such as Liaoning and Hebei, which indicates that China's carbon emissions have gradually decoupled from the degree of economic development, and the carbon emissions of economically developed regions are showing a downward trend. In terms of the time trend of regional carbon emission intensity, all regions have decreased to different degrees in the sample period, and there is a trend of further development to a lower level. It may be influenced by the economic development momentum at home and abroad in recent years, as well as the optimization and upgrading of China's energy structure and industrial structure and the innovation and progress of low-carbon technology, the energy consumption of most provinces and cities in China has reached a peak inflection point. In addition, the in-depth implementation of energy saving and carbon reduction policies in various regions has greatly reduced the carbon emission intensity of all provinces and cities in China. At present, China's provinces, cities, and regions have gradually embarked on a low-carbon and green development path, laying a solid foundation for achieving a carbon peak in 2030 and carbon neutrality in 2060.

### Exploratory spatial data analysis

#### Global spatial autocorrelation analysis

Firstly, the spatial autocorrelation between the level of scientific and technological progress and the intensity of carbon emission is tested by the overall Moran'I value. The results are listed in Table 3.

**Table 3:** Global spatial autocorrelation analysis results.

Years	2010	2011	2012	2013	2014	2015	2016	2017	2018	2019	2020
patent	0.26***	0.23**	0.25***	0.24**	0.23**	0.23**	0.22*	0.20*	0.18*	0.17*	0.17*
c	0.22**	0.22**	0.23**	0.22**	0.22**	0.21*	0.24**	0.25***	0.25***	0.30***	0.27***

***, ** and * are significant at the confidence level of 1%, 5% and 10% respectively.

Table 3 shows that the overall Moran'I of the level of scientific and technological and the intensity of carbon emission are highly significant. The Moran'I value of carbon emission intensity is between 0.21 and 0.30, and the degree of correlation has increased in recent years, indicating that there is a positive spatial correlation between the carbon emission intensity of each province. In other words, the carbon emission intensity of a province will have a positive impact on the carbon emission intensity of neighboring provinces, and this impact is becoming stronger. The Moran'I value of the level of scientific and technological progress is between 0.17 and 0.26. Although the level of scientific and technological innovation in each province shows a positive spatial autocorrelation, its positive impact reveals a downward trend year by year.

#### Local spatial autocorrelation analysis

**Figure 1:**
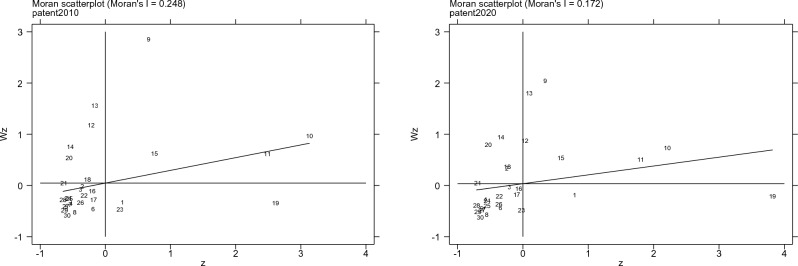
Moran scatter chart of scientific and technological progress in 2010 and 2020 (Note: The corresponding relationship between figures and provinces and cities is: 1 Beijing, 2 Tianjin, 3 Hebei, 4 Shanxi, 5 Inner Mongolia, 6 Liaoning, 7 Jilin, 8 Heilongjiang, 9 Shanghai, 10 Jiangsu, 11 Zhejiang, 12 Anhui, 13 Fujian, 14 Jiangxi, 15 Shandong, 16 Henan, 17 Hubei, 18 Hunan, 19 Guangdong, 20 Guangxi, 21 Hainan, 22 Chongqing, 23 Sichuan, 24 Guizhou, 25 Yunnan, 26 Shaanxi, 27 Gansu, 28 Qinghai, 29 Ningxia, 30 Xinjiang. Figure 2 is the same).

**Figure 2:**
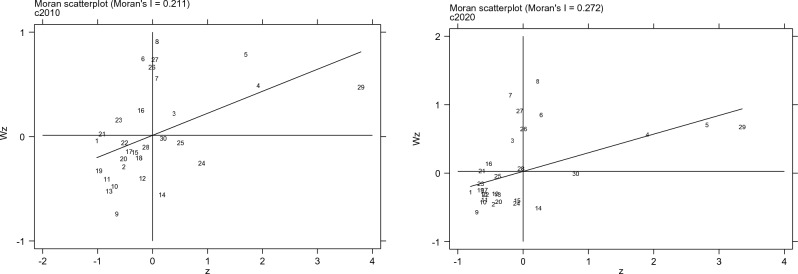
Moran scatter chart of carbon emission intensity in 2010 and 2020.

The spatial aggregation of the level of scientific and technological progress and the intensity of carbon emissions in various provinces and cities is further explored through the Moran scatter map, and the results are shown in Figs. 1 and 2 respectively.

Firstly, it analyzes the spatial aggregation of the level of scientific and technological progress in each province and city. As is shown in Fig. 1, in 2010, most provinces and cities were in a low–high and low–low concentration state, with a few in a high–high concentration state and some in a high–low concentration state. Specifically, Jiangsu, Zhejiang, and other provinces are located in high–high concentration areas, indicating that the scientific and technological progress levels of these provinces and neighboring provinces are relatively developed. Guangdong is located in a high–low concentration area, whose level of scientific and technological progress is relatively high while that of neighboring provinces is relatively low. The level of scientific and technological progress in Qinghai, Ningxia, Xinjiang, and other regions is in a low–low concentration area, revealing that the level of scientific and technological progress in these provinces and their neighboring provinces is low. Jiangxi, Guangxi, and other provinces are in low–high concentration areas, meaning that the level of scientific and technological progress of these provinces is low while the level of scientific and technological progress of their neighboring provinces is high. In general, the spatial agglomeration state of provinces and cities has changed greatly in 2020. Most provinces and cities are in low–low agglomeration areas, a few in high–high agglomeration areas, and some in high–low agglomeration areas. Specifically, Jiangsu, Zhejiang, Shanghai, and other places are in high–high concentration areas; Guangdong is in high–low concentration areas; Xinjiang, Ningxia, and other places are in low–low concentration areas. This may owe to the developed economic level of Jiangsu, Zhejiang, and Shanghai as well as its relatively-concentrated education resources and colleges, and the policies issued by local governments which are conducive to the gathering of talent. In addition, the radiation effect of big cities makes the urban scientific and technological levels of Jiangsu, Zhejiang, and Shanghai reach the forefront of China. Xinjiang and Ningxia, located in the west of China, are far behind the eastern coastal areas in terms of economic development and college education. At the same time, the local favorable policies for talent are also not as good as those in the east, resulting in a low level of science and technology in Xinjiang, Ningxia, and surrounding cities.

Then it analyzes the spatial concentration of carbon emission intensity in each province and city. As can be seen from Fig. 2, in 2010, most provinces and cities were in a low–low and low–high concentration state, with a few in a high–low concentration state and some in a high–high concentration state. Specifically, Shanxi, Inner Mongolia, and other provinces are located in high–high concentration areas, that is, the carbon emission intensity of these provinces and their neighboring provinces is relatively high. Guizhou and Yunnan are located in high–low concentration areas whose carbon emission intensity is relatively high while that of neighboring provinces is relatively low. The carbon emission intensity of Beijing and Tianjin is in the low–low concentration area, indicating that the carbon emission intensity of these provinces and their neighboring provinces are low. Liaoning, Jilin, and other provinces are in low–high concentration areas, indicating that these provinces have low carbon emission intensity while their neighboring provinces have high carbon emission intensity. There is no significant change in 2020 overall, and locally, Guizhou moves to a low–low concentration area.

### Spatial metrological analysis

#### Model selection

The Moran'I test shows that the impact of technological innovation on carbon emission intensity presents significant spatial autocorrelation. However, the Moran'I test can only test whether there is spatial autocorrelation between variables. The LM test can further test the existence of spatial autocorrelation. Therefore, the LM test is carried out on the panel data of technological innovation affecting carbon emission intensity, including LM-error test, Robust LM-error test, LM-lag test Robust LM-lag test Among them, the alternative hypothesis model of LM-error test is the spatial error autocorrelation and the spatial error moving average model. The alternative hypothesis model of LM-lag test is the spatial lag model. The Robust LM-error test and the Robust LM-lag test are amendments to the LM-error test and the LM-lag test when the model error occurs.

It can be seen from Table 4 that the p-value of the spatial error model is 0.60, which is not significant. The modified spatial error model has passed the significance level test of 5%, indicating that the residual estimated by the model has spatial autocorrelation. The *p*-value of the spatial lag model is 0.00, which is significant at the 1% level, so the spatial lag model is better than the spatial error model. Then Wald and LR tests were carried out, and both passed the 1% significance level test. The test results rejected that the spatial Durbin model would degenerate into a spatial error and the lag model, so the spatial Durbin model needs to be selected for quantitative analysis. In addition, the Hausman test results show that the statistical value of t is 24.35 and the *p*-value is 0.01, which is significant at the level of 5%, indicating that the original hypothesis that the random effect is superior to the fixed effect should be rejected, and the fixed effect model should be selected. With the estimation results of the spatial Durbin model compared and analyzed under the conditions of fixed space, fixed time, and double fixed time and space, as shown in Table 5, the R2 value and the log-likelihood of fit in the three fixed effects are both spatiotemporal double fixed coefficients are relatively large.

**Table 4:** Model inspection.

Inspection method	T-Statistics	*P* value
LM(error)	0.27	0.60
R-LM(error)	15.44	0.01
LM(lag)	11.39	0.01
R-LM(lag)	26.56	0.01
LR(ind)	23.33	0.01
LR(time)	963.18	0.00
Wald-lag	36.01	0.00
Wald-error	32.80	0.00
Hausman	24.35	0.01

**Table 5:** Fixed effect coefficient of spatial Durbin model.

Coefficient	ind(fe)	time(fe)	both(fe)
R2	0.71	0.57	0.62
Log-likelihood	− 17.91	382.97	2.51

To sum up, the SDM model with the fixed effects is selected for the regression analysis, and the regression results of SEM model and SAR model are used as a reference for comparison. Read Table 6 for the results.

**Table 6:** **.**Regression results of the spatial econometric model.

Variables	Fixed effect SDM model	SEM model	SAR model
Inpatent	− 0.20*** (0.08)	− 0.16*** (0.05)	− 0.16*** (0.05)
Inhedu	− 1.05*** (0.22)	− 1.09*** (0.21)	− 0.97*** (0.22)
rd	− 0.16** (0.09)	− 0.22*** (0.08)	− 0.19** (0.08)
urban	− 0.07*** (0.01)	− 0.02*** (0.01)	− 0.04*** (0.01)
Intec	1.49*** (0.12)	1.33*** (0.12)	1.41*** (0.12)
trand	0.19*** (0.29)	− 0.36*** (0.26)	− 0.26*** (0.27)
Log-likelihood	− 17.91	− 31.21	− 35.54
R2	0.71	0.68	0.67
Observations	330	330	330
Number of code	30	30	30

The values in brackets in the table are the Z statistic values of the spatial econometric model, the same as below.

Standard errors in parentheses ***p < 0.01, **p < 0.05, *p < 0.1.

It can be seen from Table 6 that under the SDM model with fixed effects, the coefficient of the technological innovation level is significantly negative at the level of 1%, which indicates that the technological innovation level has a strong inhibitory effect on the growth of carbon emission intensity. But will this relationship change due to different model choices? Therefore, the regression results of the SEM model and SAR model introduced in this paper are compared with the SDM model under the double-fixed effect. From Table 6, we can find that the coefficient of technological innovation level is also significantly negative under the SEM model and SAR model. In addition, through comparison, it is found that the positive and negative regression coefficients of each model are relatively consistent and the significant number difference is not large, so it is more reasonable to select the SDM model with a double fixed effect model.

#### Analysis of spatial spillover effect

The analysis of the spatial impact of the level of technological innovation on carbon emission intensity requires a partial differential decomposition of the SDM model to examine the direct and indirect effects of the variables. The specific direct and indirect effects under the Fixed effect SDM model are listed in Table 7.

**Table 7:** Direct and indirect effects under the Fixed effect SDM model.

Variables	Direct effect	Indirect effect	Total effect
Inpatent	− 0.20*** (0.08)	− 0.04 (0.09)	− 0.24*** (0.06)
Inhedu	− 1.11*** (− 0.25)	− 1.31*** (0.44)	− 2.42*** (0.45)
rd	− 0.15** (0.08)	− 0.05 (0.15)	− 0.20 (0.16)
urban	− 0.07*** (0.01)	0.09*** (0.02)	0.02** (0.02)
Intec	1.49*** (0.11)	− 0.08 (0.26)	1.41*** (0.29)
trand	0.23*** (0.29)	0.27* (0.57)	0.49*** (0.59)
Log-likelihood	− 17.91	− 17.91	− 17.91
R2	0.71	0.71	0.71
Observations	330	330	330
Number of code	30	30	30

It can be seen from Table 7 that the direct effect of technological innovation level on carbon emission intensity is significantly negative, indicating that the improvement of technological innovation level in a province has a significant inhibitory effect on local carbon emission intensity; the indirect effect is positive and the result is not significant, which indicates that the improvement of local technological innovation level has little impact on the carbon emission intensity of neighboring provinces and cities, but the overall effect is negative. Despite a rebound effect on carbon emissions concerning the level of technological innovation in the short term^40^, the intensity of carbon emissions is not only related to carbon emissions but also related to a certain level of economic development. Therefore, in the long run, the improvement of technological innovation level will still inhibit the increase of carbon emission intensity. The direct effect, indirect effect, and total effect of a national cultural level are significantly negative at the level of 1%, which indicates that the improvement of a national cultural level not only has a significant inhibition effect on local carbon emission intensity but also inhibits the carbon emission intensity of neighboring provinces and cities. In October 2022, the Chinese government proposed that promoting green and low-carbon economic and social development is the key link to achieving high-quality development. The improvement of the national cultural level can promote national comprehensive quality and environmental awareness, and better implement the initiatives put forward by the Chinese government in life, so as to consciously advocate low-carbon life^41^. The direct effect of R&D investment intensity on carbon emission intensity is significantly negative, indicating that the improvement of local R&D investment intensity will have a restraining effect on carbon emission intensity to a certain extent. The indirect effect and total effect are not significant enough, indicating that the intensity of local R&D investment only has an impact on the intensity of carbon emissions in the province and city. According to the report on China's investment in science and technology in 2021, China invested a total of 2795.63 billion yuan in research and experimental development, an increase of 356.32 billion yuan over the previous year, with an annual increase of 14.6%^42^. The intensity of R&D investment is the key factor to improve the level of scientific and technological innovation, so the increase of R&D investment intensity will indirectly inhibit the intensity of carbon emissions. The direct and total effects of urbanization level are significantly negative at the level of 1%, while the indirect effects are not significant enough, which shows that the improvement of urbanization level in a province has a significant inhibitory effect on the carbon emission intensity of the province, but has little impact on the neighboring provinces and cities. From 2010 to 2020, China's urbanization rate rose from 49.68 to 63.89%. The acceleration of urbanization will lead to the expansion of the existing city scale and the emergence of a number of new cities. Accordingly, the demand for energy-intensive industries such as steel and cement will also be more vigorous, which will inevitably lead to a significant increase in China's carbon emissions^43,44^. However, with the increase in urban population and the explosion of energy-intensive industries, the huge economic benefits brought by the increase in the urbanization rate cannot be denied.

As the ratio of carbon emissions to GDP, the carbon emission intensity has a certain inhibitory effect on the carbon emission intensity when the economic benefits brought by the urbanization process exceed the carbon emissions generated by itself. The direct effect of total energy consumption on carbon emission intensity is positive and highly significant, which shows that the total energy consumption mainly based on fossil energy plays a huge role in promoting the improvement of local carbon emission intensity. As a typical country with coal consumption as its main source, China's coal consumption accounted for about 64% of China's total energy consumption in 2015. It can be said that China's rapid economic growth is at the cost of huge coal energy consumption^45,46^. In recent years, the proportion of coal energy consumption in China's total energy consumption has declined, but it still exceeds 50%. In addition to the proportion of other fossil fuels, it is still a huge source of continuous growth in carbon emissions. Therefore, when the total energy consumption increases year by year, China's GDP growth slows, which will inevitably lead to an increase in the intensity of carbon emissions. The direct and indirect effects of China's foreign trade level on carbon emission intensity are significantly positive in the model regression results, indicating that the level of foreign trade not only promotes the improvement of local carbon emission intensity but also promotes the improvement of carbon emission intensity in neighboring provinces and cities. China has already realized the transformation of its export product structure from primary agricultural products such as agricultural and sideline products to major industrial products, with steel and cement as major^47^. In recent years, the increase in China's export volume has also indirectly promoted the production of these energy-intensive industries, followed by the breeding of a large amount of carbon dioxide emissions, leading to an increase in carbon emission intensity.

#### Robustness test based on different regions

Because the regression results of spatial econometric models are largely affected by regional factors, this paper divides China into eastern, central, and western regions, and tests the robustness of the models for each regional regression. The results are shown in Table 8.

**Table 8:** Results of SDM model robustness test based on different regions.

Variables	Eastern regions	Central regions	West regions
	Direct effect		
Inpatent	− 0.25*** (0.05)	− 0.19*** (0.08)	− 0.37*** (0.20)
Inhedu	− 0.27* (0.20)	− 0.06 (0.33)	− 1.36*** (0.496)
rd	− 0.19*** (0.04)	− 0.12 (0.15)	− 0.45*** (0.26)
urban	− 0.05*** (0.01)	0.01 (0.02)	− 0.18*** (0.03)
Intec	0.81*** (0.09)	0.95*** (0.18)	1.65*** (0.30)
trand	0.25*** (0.18)	1.88 (1.29)	2.17*** (1.28)
Indirect effect			
Inpatent	0.05 (0.05)	− 0.26*** (0.09)	0.38*** (0.24)
Inhedu	0.47* (0.27)	0.62 (0.63)	− 1.47*** (1.05)
rd	0.00 (0.06)	− 0.03 (0.19)	− 0.89 (0.59)
urban	0.02** (0.01)	− 0.01 (0.03)	0.29*** (0.04)
Intec	− 0.06 (0.12)	− 1.01*** (0.35)	1.07 (0.74)
trand	0.41*** (0.22)	6.87*** (1.96)	1.29 (2.73)
Total effect			
Inpatent	− 0.19*** (0.03)	− 0.46*** (0.09)	− 0.75*** (0.20)
Inhedu	0.74** (0.31)	0.56 (0.90)	− 2.83*** (1.08)
rd	− 0.19** (0.08)	− 0.15 (0.23)	− 1.34** (0.66)
urban	− 0.03*** (0.01)	0.00** (0.03)	− 0.11*** (0.04)
Intec	0.75*** (0.11)	0.06*** (0.49)	2.72*** (0.79)
trand	− 0.65** (0.19)	8.75*** (2.71)	− 3.46** (− 2.82)

Standard errors in parentheses ***p < 0.01, **p < 0.05, *p < 0.1.

It can be seen from the results in Table 8 that the direct effect of technological innovation level in the eastern, central, and western regions is significantly negative, consistent with the above conclusions, and the control variables have not changed significantly. This proves that the conclusion of this paper is reliable.

### Threshold effect analysis

#### Threshold effect test

This paper takes the level of economic development as the threshold variable to explore the nonlinear impact of technological innovation level on carbon emission intensity under different economic development levels. Firstly, the number of threshold variables needs to be determined. The test results are listed in Table 9.

**Table 9:** Threshold effect test.

Variables	Number of thresholds	F value	*P* value	Estimated value of threshold	95% confidence interval		BS times
Economic development level	Single threshold	97.39	0.00	2.37	2.32	− 2.37	300
	Double threshold	43.98	0.01	2.54 1.71	2.44 1.69	− 2.54 − 1.72	300
	Triple threshold	44.66	0.28	2.26	2.21	− 2.27	300

It can be seen from Table 9 that at different levels of economic development, both the single threshold and the double threshold have passed the 1% significance level test, while the triple threshold has not passed the significance level test. Therefore, there are single and double thresholds for the impact of technological innovation level on carbon emission intensity. The estimated value of the single threshold of economic development level is 2.37 and the estimated value of the double threshold of economic development level is 2.54 and 1.71.

#### Threshold regression results

Table 10 shows the regression results with the level of economic development as the threshold variable.

**Table 10:** Threshold regression results.

Variables	Economic development level
Inhedu	− 0.48*** (0.15)
rd	− 0.19** (0.08)
Intce	0.97*** (0.13)
urban	− 0.96*** (0.27)
trand	0.04 (0.05)
Inpgdp ≤ ${\upgamma }_{1}$	− 0.10*** (0.03)
${\upgamma }_{1}$≤Inpgdp ≤ ${\upgamma }_{2}$	− 0.15*** (0.03)
Inpgdp ≥ ${\upgamma }_{2}$	− 0.21*** (0.03)
Constant	− 2.92** (1.19)
Observations	330
Number of code	30
R-squared	0.83

Robust standard errors in parentheses ***p < 0.01, **p < 0.05, *p < 0.1.

With the level of economic development as the threshold variable, there is a double threshold between the level of technological innovation and the intensity of carbon emissions, and the impact coefficients are both negative but slightly different. When the economic development level is lower than the first threshold value of 2.37, the impact coefficient is − 0.10; after crossing the first threshold, the influence coefficient is − 0.15, indicating that the inhibition effect is enhanced at this time; after crossing the second threshold of 2.54, the influence coefficient becomes − 0.21, and the inhibition effect is further strengthened. It indicates that when the level of economic development is taken as the threshold variable, there is an obvious nonlinear relationship between the level of technological innovation and the intensity of carbon emissions, and the inhibition effect increases with the improvement of the level of economic development. It can be seen from Table10 that under different levels of economic development, both the single threshold and the double threshold have passed the test of 1% significance level, while the triple threshold has not passed the significance level test. Therefore, there are single and double thresholds for the impact of technological innovation level on carbon emission intensity, respectively. The single “Economic Development Level Threshold” threshold is estimated at 2.37 and the double “Economic Development Level Threshold” threshold is estimated at 2.54 and 1.7066. The threshold variable economic development level in this paper is measured by GDP per capita, and the two thresholds shown in the threshold regression results indicate that when China's per capita GDP level reaches the two critical values of 2.37 and 1.71, the impact of technological innovation on carbon emission intensity will change. From the point of view of time, after 2012, although China's economy is developing rapidly, the economic form is promising, but too rapid industrialization, industrialization has also brought ecological impact, at this time China's overall economic level reached the first threshold, technological innovation began to show the inhibition effect on carbon emissions, the concept of “scientific and technological carbon reduction” became popular, and the Chinese government and society paid more attention to the protection of the ecological environment. Around 2017, after the 19th Congress of the Communist Party of China, the environmental problems caused by China's rapid economic growth have been paid more and more attention, and the concepts of “green” and “low-carbon” have been put forward. At the same time, China has entered a stage of relative economic prosperity, the government proposes to develop a high-quality economy, at this time the Chinese government and all sectors of society have a certain amount of “pockets”, so more vigorously strengthen scientific and technological investment. At this time, China's economic development level has reached around the second threshold, and the level of scientific and technological innovation has been continuously improved at this stage, which has brought about the corresponding impact of increasing carbon emission intensity inhibition. When the level of economic development is in the third range, the inhibition effect is the largest. Economic development provides a material basis for scientific and technological innovation, promotes the output of scientific and technological innovation, improves the efficiency of innovation transformation, and creates a good innovation environment. It is the high-quality development of China's economic level in recent years that has led to major breakthroughs in green and low-carbon technologies. Green and low-carbon technological innovation is not only an important part of the new round of global industrial revolution and technological change, but also an important support for China to cope with climate change, promote high-quality development and achieve the goal of building a beautiful China^48^. Therefore, the economic level plays an important role in the level of technological innovation and the intensity of carbon emissions. With the growth of the economic level year by year, the inhibition of the level of technological innovation on the intensity of carbon emissions is becoming more and more significant.

#### Threshold interval classification analysis

From the threshold regression results, it can be seen that the level of technological innovation has an obvious nonlinear impact on the carbon emission intensity under the level of economic development. This paper takes 2020 as an example to analyze the corresponding threshold range of each province, city, and region, in order to provide targeted suggestions for each province and city.

It can be seen from Table 11 that when the level of economic development is taken as the threshold variable, the optimal range for reducing carbon emission intensity of technological innovation level is the third range. In 2020, Beijing, Tianjin, Shanghai, Jiangsu, Zhejiang, Guangdong, and other places are in the optimal range, while Hebei, Shanxi, Jilin, and other places are not in the optimal range.

**Table 11:** Threshold range of provinces, regions and cities in 2020.

Variables	First interval	Second interval	Third interval
Economic development level	No one	Hebei, Shanxi, Jilin, Heilongjiang, Jiangxi, Guangxi, Guizhou, Yunnan, Gansu, Qinghai, Ningxia	Beijing, Tianjin, Inner Mongolia, Liaoning, Shanghai, Jiangsu, Zhejiang, Anhui, Fujian, Shandong, Henan, Hunan, Hubei, Guangdong, Hainan, Chongqing, Sichuan, Shaanxi, Xinjiang

## Conclusions and suggestions

This paper takes the panel data of 30 provinces and cities in China from 2010 to 2020 as the research object, measures and analyzes the carbon emission intensity and the scientific and technological levels, and adds control variables to investigate the spatial spillover effect of technological innovation level on carbon emission intensity, and further adds threshold variables to investigate the nonlinear impact of technological innovation level on carbon emission intensity. The study draws the following conclusions: first, from 2010 to 2020, the carbon emission intensity and the scientific and technological levels of 30 provinces and cities in China showed an obvious spatial–temporal change trend. From the perspective of time, the carbon emission intensity of most provinces and cities in 2020 has decreased compared with that in 2010, which indicates that China has made certain achievements in energy conservation, emission reduction, and low-carbon policies in recent years. The level of technological innovation in all provinces and cities has improved, which shows that China's science and technology have developed rapidly in recent years, and the strategy of invigorating the country through science and education has been perfectly implemented. From the perspective of space, the intensity of carbon emissions and the level of technological innovation in China's provinces and cities show significant heterogeneity. The provinces and cities with high carbon emissions are mainly concentrated in the central and western regions, while the provinces and cities with high levels of technological innovation are mainly concentrated in the eastern regions. Secondly, the spatial spillover regression results show that the level of technological innovation has a significant inhibitory effect on the local carbon emission intensity. The improvement of the national cultural level will inhibit the carbon emission intensity of local and neighboring provinces and cities. The intensity of R&D investment and the average urbanization water has a significant impact on the local carbon emission intensity inhibition. The total amount of energy consumption and the level of foreign trade dominated by fossil energy consumption are important factors to improve the intensity of carbon emissions. Thirdly, the panel threshold regression results show that there is a nonlinear effect between the level of technological innovation and the intensity of carbon emissions, and there is a double threshold of economic development level between them. When the level of economic development is in the third range, the level of technological innovation has the greatest inhibiting effect on the intensity of carbon emissions. Compared with the existing literature, this paper has two innovation. Firstly, the existing literature research on the relationship between technological innovation level and carbon emissions mostly focuses on carbon emission efficiency and overall carbon emissions, while this paper introduces economic factors to replace previous research objects with carbon emission intensity. Secondly, in terms of research methods, this paper first discusses the linear relationship between technological innovation and carbon emission intensity by spatial econometric methods, and then introduces a threshold model to explore the nonlinear relationship between the two that has been ignored in previous literature.

Based on the above research findings, the following suggestions are put forward: Firstly, the central and western regions should vigorously develop low-carbon technologies, introduce scientific and technological talents, and be prepared to undertake the happy technology industry in the east, increasing support for enterprises to independently research and develop low-carbon technologies^49^. Of course, in the specific implementation process, the western region may face funding shortages, inadequate infrastructure, and a lack of follow-up talent management in talent introduction. At that time, the local government should continuously innovate favorable policies for talent introduction and improve and optimize existing policies, provide significant material and financial support, and do a good job in managing and handling the aftermath of the arrival of a series of talents. The government should also establish specialized agencies to cope with the upcoming large number of talents. Secondly, achieving the goals of carbon neutrality and carbon peaking requires incorporating spatial spillover effects into the deployment of energy conservation and emission reduction strategies, conducting regional joint governance of environmental pollution^50^, building a coordinated mechanism for energy conservation and emission reduction policies that are comprehensive and inclusive of individual cities, and forming regional docking plans in policies such as technology innovation capability enhancement plans, new energy green industry support, and green technology subsidies. From the perspective of practical implementation difficulties, the main possibility lies in how to achieve regional coordination and linkage, and how to formulate policies that are not only in line with the ecological and environmental protection of the local area but also take into account the economic development of the region. In response to this issue, this article suggests that the role of “think tanks” in various regions can be utilized to collect relevant ideas from leaders in the region, and relevant information can be published on the Internet to widely solicit public opinions and achieve unity as much as possible. Thirdly, various provinces in China should increase investment in scientific research and support enterprises and universities in their research and innovation in energy conservation and emission reduction. Of course, there will also be related issues such as talent shortage during the implementation process. At this time, it is necessary to increase the incentives for talent introduction, and do a good job in various follow-up work to form a long-term mechanism that continuously attracts talents. Fourthly, various provinces and cities in China need to gradually reduce fossil energy consumption, increase the utilization rate of new energy^51^, implement carbon emission indicators in various regions, increase carbon tax intensity, and impose penalties for illegal emissions^52^. During the implementation process, it may have a certain impact on some industrial production. The development and utilization of new energy have not reached the level of comprehensive substitution for fossil energy, and industrial production mainly relies on the consumption of fossil energy. Therefore, in order to solve this problem, we can only increase research on the development and utilization of new energy in the future. In addition, the research findings and policy recommendations of this article are equally applicable to developing countries facing the same problems as China. In addition, from the perspective of economic development level, there are still areas such as Guizhou, Yunnan, Gansu, and Qinghai that have not reached the optimal range. In the future, we need to seize economic development opportunities and accelerate the pace of regional economic development.

## Research deficiencies and future prospects

There are several shortcomings in the research of this paper, first, the sample size is small, which may lead to bias in the research conclusion and lack of certain universality; Second, the academic community has different standards for the measurement of related indicators, for example, there are different opinions on the measurement methods of technological innovation level, and the data obtained by different measurement methods may be very different, which also has a certain impact on the research results; Third, in terms of research methods, this paper only uses a weight matrix for research, which may lead to the instability of the research results. Therefore, in order to eliminate the influence of these factors as much as possible, subsequent studies need to expand the sample size, compare the results obtained by a variety of index measurement methods, and finally select the optimal terms, and introduce time and space weight matrices to further explore the robustness of the research results.

## Data Availability

The data presented in this study are available on request from the corresponding author.
